# Capability Assessment for Diet and Activity (CADA) and Its Influencing Factors Among Healthcare Workers in the Jazan Region, Saudi Arabia, 2026: A Cross-Sectional Study

**DOI:** 10.3390/healthcare14111530

**Published:** 2026-06-01

**Authors:** Yahya H. Almalki, Amal J. Alfaifi, Abdullah A. Mosawa, Abdulrahman M. Mahzara, Mohammed H. Abutaleb

**Affiliations:** 1 Department of Family Medicine, Jazan Health Cluster, Jazan 82732, Saudi Arabia; ajh6666@hotmail.com (A.J.A.); almosawa1@hotmail.com (A.A.M.); 2 Faculty of Medicine, Jazan University, Jazan 86555, Saudi Arabia; mhzrybdalrhmn477@gmail.com; 3 BCACP Pharmaceutical Care Department, King Fahd Central Hospital in Jazan, Jazan Health Cluster, Jazan 82666, Saudi Arabia; abutaleb33@gmail.com

**Keywords:** healthcare workers, capability assessment for diet and activity (CADA), physical activity, diet, Jazan health cluster

## Abstract

**Highlights:**

**Public health relevance—How does this work relate to a public health issue?**

- Healthcare workers are a key target group for lifestyle promotion, as their capability to adopt healthy behaviors may influence both their own health and their role in promoting healthy behaviors.
- Assessing perceived capability for diet and physical activity in Jazan provides insight into workplace-related constraints relevant to chronic disease prevention.

**Public health significance—Why is this work of significance to public health?**

- Healthcare workers in Jazan demonstrated moderate perceived overall capability, with higher perceived capability for diet than for physical activity.
- Lower perceived capability observed among primary healthcare center staff and non-physician professionals highlights perceived disparities within the healthcare workforce.

**Public health implications—What are the key implications or messages for practitioners, policy makers, and/or researchers in public health?**

- Workplace-based interventions targeting time constraints, physical activity barriers, and access to healthy food may improve capability among healthcare workers.
- Primary healthcare settings may require focused wellness strategies to enhance healthy lifestyle capability and reduce barriers.

**Abstract:**

**Background**: Adopting a healthy lifestyle through a balanced diet and regular physical activity is essential for chronic disease prevention, but healthcare workers face occupational constraints that may limit such behaviors. This study assessed perceived capability for healthy diet and physical activity among healthcare workers in the Jazan region of Saudi Arabia using the Capability Assessment for Diet and Activity (CADA) instrument and examined associated factors. **Methods**: A cross-sectional analytical study was conducted in 2026 in governmental healthcare facilities in the Jazan Health Cluster. A structured electronic questionnaire collected sociodemographic, occupational, and health-related data alongside the 34-item CADA. Total, Diet and Physical Activity CADA scores (1–5) were analyzed using descriptive statistics and multivariable ordinary least squares regression adjusted for sex, education, profession, and workplace; standardized coefficients and Cohen’s f^2^ were reported. **Results:** A total of 601 healthcare workers participated. Internal consistency was good (Cronbach’s α = 0.84 for the full scale). Mean Total CADA was 3.28 ± 0.80 (scale midpoint 3.0); perceived Diet capability (3.45 ± 0.85) was higher than perceived Physical Activity capability (3.11 ± 0.85). Female sex was independently associated with lower Physical Activity CADA (β = −0.16; 95% CI −0.32 to −0.01; *p* = 0.042). Bachelor’s and board/doctoral qualifications were associated with higher Total CADA (β = 0.20; 95% CI 0.02 to 0.38; *p* = 0.026 and β = 0.33; 95% CI 0.07 to 0.58; *p* = 0.013, respectively). Compared with hospital-based participants, those in primary healthcare centers had lower Total (β = −0.19; 95% CI −0.32 to −0.05; *p* = 0.007), Diet (β = −0.17; 95% CI −0.31 to −0.02; *p* = 0.024) and Physical Activity (β = −0.21; 95% CI −0.35 to −0.06; *p* = 0.006) CADA scores. Effect sizes were small (|β*| ≤ 0.16; R^2^ = 0.076–0.082; Cohen’s f^2^ = 0.08–0.09). **Conclusions:** As CADA captures perceived capability, these findings reflect self-perception rather than objectively measured behavior; longitudinal studies combining CADA with validated behavioral instruments are warranted to clarify whether perceived capability translates into actual dietary and physical-activity behaviors in healthcare workers, and to evaluate whether workplace-based interventions targeting time pressure and access to supportive environments improve both perceived capability and measured behavior.

## 1. Introduction

Regular physical activity and a balanced diet are foundational lifestyle behaviors: they are linked to lower cardiovascular, metabolic and oncological morbidity, better cognitive function and lower levels of anxiety and depression, whereas sedentary behavior and unhealthy diets are central drivers of the global rise in non-communicable diseases (NCDs) such as cardiovascular disease, diabetes and certain cancers [1,2,3,4]. Because both behaviors are modifiable, lifestyle promotion remains a cornerstone of NCD prevention, and primary healthcare providers, through frequent patient contact, counselling and follow-up, are central to its delivery [5,6,7,8].

However, the effectiveness of such counselling depends on providers’ knowledge and their perceived capability to adopt healthy behaviors, as knowledge alone is insufficient to sustain lifestyle change when structural or environmental barriers are present [9,10]. Lifestyle-related risk factors are particularly prevalent in countries undergoing rapid social and economic transition. In Saudi Arabia, national surveys report obesity prevalence of around 24.7% in 2020 (down slightly from 25.6% in 2018 and 28.7% in 2013) [11,12,13], while physical-inactivity estimates range widely across studies (4–44.5% in Saudi Arabia and 34.2–96.9% across Arab countries) owing to methodological differences [14,15]. Together with the rising availability of energy-dense foods and limited opportunities for physical activity, these patterns place a substantial burden on the healthcare system [16,17,18,19,20,21,22].

Healthcare workers (HCWs) represent a unique population within the broader public health landscape. As frontline providers, they are expected to model healthy behaviors and serve as credible sources of lifestyle advice. However, HCWs often face occupational barriers that hinder their ability to adopt and maintain healthy behaviors. Workload, shift duties, sleep disruption, and limited access to healthy food during work hours may negatively affect their diet and physical activity patterns. Long working hours and high job demands can lead to fatigue, stress, and reduced motivation to engage in physical activity. These barriers may not only compromise HCWs’ own health but also influence their confidence and effectiveness in counseling patients about lifestyle modification. Evidence suggests that healthcare providers who practice healthy behaviors themselves are more likely to counsel patients effectively and to be perceived as credible by those they serve.

Assessing capability provides insight into lifestyle adoption beyond knowledge alone. Capability reflects the extent to which individuals perceive themselves as able to engage in healthy behaviors, considering personal, social, and environmental factors. The Capability Assessment for Diet and Activity (CADA) instrument offers a structured approach to evaluating these factors by examining domains such as opportunity, barriers, knowledge, time pressure, and social support. A Saudi study using the CADA instrument demonstrated substantial interregional variation in capability, highlighting the need for region-specific assessment [23]. Such variation may reflect differences in cultural norms, workplace environments, socioeconomic conditions, and access to supportive resources.

The Jazan region presents a particularly important context for studying HCWs’ perceived capability. Located in southwestern Saudi Arabia, Jazan has a diverse population and a rapidly expanding healthcare system. The region faces a high burden of chronic diseases, including obesity, diabetes, and hypertension, which underscores the importance of preventive health strategies. Therefore, understanding the perceived capability of HCWs in this region to engage in healthy diet and physical activity is essential for designing effective workplace wellness programmes and strengthening the role of HCWs in lifestyle counselling.

The three constructs of perceived capability, self-efficacy and actual behavior are conceptually distinct. Self-efficacy refers to a person’s confidence in their ability to perform a specific behavior under specific conditions; actual behavior refers to observable or measurable dietary intake or physical activity; and perceived capability, as operationalized by the CADA instrument, captures a broader appraisal of personal, social and environmental factors that enable or constrain healthy diet and physical activity. The present study assesses perceived capability and does not measure self-efficacy or actual behavior.

Therefore, this study aimed to assess capability for healthy diet and physical activity and to examine its sociodemographic, occupational, and health-related correlates among healthcare workers in the Jazan region of Saudi Arabia in 2026. By identifying factors that influence capability, the study provides evidence to guide targeted interventions that support HCWs in adopting healthier behaviors and enhancing their role in promoting lifestyle modification among patients.

## 2. Materials and Methods

### 2.1. Study Design

A cross-sectional analytical study was conducted in 2026 among healthcare workers in the Jazan region of Saudi Arabia to assess capability for healthy diet and physical activity and the factors associated with it.

### 2.2. Study Setting

This study was conducted in governmental healthcare institutions, including hospitals and primary healthcare centers (PHCs), in the Jazan health cluster. The questionnaire captured participants’ workplace setting (PHC, hospital, or administrative) and work schedule (routine vs. shift work) (Supplementary Table S1).

### 2.3. Study Population

The study population comprised healthcare workers employed in governmental healthcare facilities during the study period, including physicians, dentists, nurses, and allied health professionals involved in direct clinical or patient-related work. Occupational categories included several clinical disciplines, such as medicine, dentistry, radiology, laboratory services, pharmacy, physiotherapy, respiratory therapy, clinical nutrition, public health, and health education.

### 2.4. Inclusion Criteria

Participants were eligible if they were aged 18 years or older, were currently employed in a governmental healthcare facility in the Jazan region, were engaged in clinical or patient-related activities, and provided electronic informed consent before participation.

### 2.5. Exclusion Criteria

Participants were excluded if they were on long-term leave during the data collection period or submitted incomplete questionnaires missing key outcome or covariate data required for the planned analyses.

### 2.6. Sample Size and Sampling Technique

The minimum required sample size was calculated using the Raosoft formula with a 95% confidence level and a 5% margin of error. Because no previous estimate of CADA capability among HCWs in the Jazan region was available, a conservative expected proportion of 50% was assumed, yielding a minimum sample size of 384 participants. To account for possible non-response and incomplete questionnaires, 10% was added, resulting in a target sample of 420 participants.

The sampling process utilized a stratified convenience approach, necessitated by the high clinical workload and workforce constraints within the Jazan Health Cluster. Evidence from the Jazan region indicates that a substantial proportion of healthcare workers experience high levels of work-related stress (66.3%). Previous literature suggests that factors such as workload and time pressure are important contributors to occupational stress [24]. In addition, recent studies from Jazan hospitals have reported a high prevalence of burnout among healthcare workers, particularly nurses and intensive care unit staff. This has been linked to workplace stressors such as high workload, long working hours, and staffing shortages [25,26]. Given these conditions, strict random sampling of individuals would result in a high non-response rate and potential disruption to essential clinical services. Therefore, a more pragmatic sampling approach was adopted. This approach is consistent with previous studies conducted in the Jazan region, where convenience sampling was used in healthcare settings [24].

Accordingly, a stratified convenience sampling approach was used to improve representation across key professional groups and healthcare settings. The sample was stratified by profession (physicians/dentists, nurses, and allied health professionals) and workplace setting (hospitals and PHCs). Within these strata, eligible HCWs were invited to participate using convenience sampling during the study period. Recruitment continued until the end of data collection. A total of 601 questionnaires were received, and all submitted questionnaires contained complete data for the variables included in the present analysis; therefore, all 601 questionnaires were included in the descriptive, bivariate, and multivariable analyses.

### 2.7. Recruitment and Data Collection Procedure

Eligible HCWs working in governmental healthcare facilities in the Jazan Health Cluster were approached during the study period. Data collection was completed before Ramadan 2026, and data analysis was initiated on 6 February 2026, prior to the fasting month. The Jazan Health Cluster employs approximately 13,880 healthcare workers across hospitals, primary healthcare centers, and administrative units (an exact public census is not available; this figure was obtained from the Cluster’s human-resources department). The questionnaire link was distributed through official workplace communication channels and via WhatsApp groups maintained by the Cluster and by departmental coordinators. The link was estimated to reach approximately 800 eligible HCWs. A total of 601 valid responses were received, corresponding to an approximate response rate of 75%. Because the link could be forwarded within WhatsApp groups, the denominator is approximate and the response rate should be interpreted as an estimate.

The achieved sample comprised 43.1% physicians/dentists, 32.0% nurses and 24.9% allied health professionals, distributed across hospitals (49.4%) and primary healthcare centers (47.3%); this composition mirrors the known professional and workplace distribution of HCWs in the Jazan Health Cluster, although physicians and hospital-based staff are likely to be modestly over-represented relative to a fully random sample.

### 2.8. Data Collection Instrument

Data were collected using a structured, self-administered electronic questionnaire composed of three sections. The Capability Assessment for Diet and Activity (CADA) instrument used in the present study was adapted from a previously published and validated instrument [27].

The questionnaire included the following sections:

1. Sociodemographic and occupational variables: Age, sex, marital status, nationality, educational level, occupation, workplace, and work schedule.
2. Health and lifestyle variables: Self-reported weight and height for body mass index (BMI) calculation, self-rated health, chronic physical illness, chronic psychological illness, smoking status, physical activity frequency, current diet status, and whether participants advised patients or family members about healthy lifestyle behaviors.
3. Capability Assessment for Diet and Activity (CADA): A 34-item instrument assessing capability-related factors related to healthy eating and physical activity. Each item was scored on a 5-point Likert scale ranging from 1 (almost never) to 5 (almost always).

No validated dietary intake instrument (e.g., food frequency questionnaire) or objectively measured physical activity tool (e.g., accelerometer, IPAQ) was administered. The CADA instrument captures perceived capability to engage in healthy diet and physical activity behaviors rather than the behaviors themselves; accordingly, the findings of this study reflect self-perceived capability and not objectively measured dietary intake or physical activity.

### 2.9. CADA Domains and Scoring

The CADA instrument [27] includes 34 items grouped into the following domains: convenience/cost (items 1–7), neighborhood opportunity (items 8–12), barriers (items 13–17), knowledge (items 18–20), time pressure (items 21–25), family support (items 26–28), husband/wife (previously spouse/partner) support (items 29–31), and nonfamily support (items 32–34) [23,27]. Items were scored on a 5-point Likert scale; following standard practice for multi-item Likert instruments [27], composite scores were calculated as item means, and negatively worded items (barriers and time pressure) were reverse-coded prior to score calculation so that higher composite values consistently represent greater perceived capability.

Scores were calculated as item means on the 1–5 scale, with higher scores indicating greater perceived capability. The Total CADA score was the mean of all 34 items. The Diet CADA composite was the mean of the diet-relevant items (1, 4–7 for diet opportunity; 14, 15, 17 for diet barriers; 18–20 for diet knowledge; 22–24 for diet time). The Physical Activity CADA composite was the mean of the physical-activity-relevant items (2–3 for convenience; 8–12 for neighborhood opportunity; 13 and 16 for physical activity barriers; 21 and 25 for physical activity time). Items 13–17 (barriers) and 21–25 (time pressure) are framed as obstacles; these are reverse-coded prior to score calculation so that, in every composite, higher values represent greater perceived capability and fewer perceived constraints.

#### Psychometric Evaluation in the Present Sample

Because the CADA instrument was originally developed for the general population and not specifically validated in healthcare workers, we evaluated its internal consistency in the present sample before using the scores in inferential analyses. Cronbach’s alpha was computed for the overall 34-item scale and for each of the eight domains (convenience/cost, neighborhood opportunity, barriers, knowledge, time pressure, family support, husband/wife support, and nonfamily support). Corrected item-total correlations were inspected to identify any items contributing poorly to their domain. Negatively worded items were reverse coded before all reliability analyses. In addition, given the inclusion of unmarried participants (27.1%), the applicability of the husband/wife support domain (items 29–31) was assessed by repeating the descriptive and multivariable analyses with the Total CADA score recalculated after excluding this domain (sensitivity analysis).

### 2.10. Statistical Analysis

Data were analyzed using Stata version 15. Descriptive statistics were presented as means and standard deviations for continuous variables and frequencies and percentages for categorical variables. All 601 complete questionnaires were included in the descriptive analyses.

The distribution of the continuous CADA composite scores (Total, Diet and Physical Activity) was assessed using visual inspection of histograms and Q–Q plots, together with the Shapiro–Wilk test. The composite scores approximated a normal distribution (skewness within ±1 and kurtosis within ±2 for all three composites), supporting the use of parametric tests (independent-samples *t*-test, one-way ANOVA and OLS regression) in the subsequent analyses.

For bivariate analyses, independent-samples *t*-tests were used for variables with two categories, whereas one-way analysis of variance (ANOVA) was used for variables with more than two categories. Bonferroni post hoc comparisons were performed where appropriate.

Multivariable ordinary least squares (OLS) regression models were constructed to identify independent predictors of Total CADA, Diet CADA, and Physical Activity CADA scores. Based on conceptual relevance, consistency with the bivariate findings, and model parsimony, sex, education level, occupation, and workplace were included as covariates in the adjusted models. These variables were selected because they represented the main sociodemographic and occupational characteristics, most plausibly associated with capability.

OLS regression was selected because of the dependent variables’ (Total CADA, Diet CADA, Physical Activity CADA) mean composite scores calculated across multiple Likert items and we approximated a continuous distribution; ordinal models are more appropriate for single-item Likert responses than for averaged multi-item composites. To confirm robustness, individual-item analyses were repeated using ordinal logistic regression for each composite’s highest-loading items, and the direction and significance of the main predictors were consistent with the OLS findings. For each OLS model, the following diagnostics were performed: visual inspection of residual-versus-fitted and Q–Q plots; the Breusch–Pagan test for heteroskedasticity; variance inflation factors (VIF) for multicollinearity (threshold > 5 considered indicative); and Cook’s distance for influential observations. Covariates entered the multivariable model based on (i) *a priori* conceptual relevance to perceived capability, (ii) statistical significance in bivariate analyses (*p* < 0.10) and (iii) the avoidance of conceptual overlap; this parsimonious approach was chosen given the modest signal-to-noise ratio and to minimize overfitting.

To convey the practical magnitude of the associations rather than statistical significance alone, standardized regression coefficients (β*) were computed in addition to unstandardized β, and Cohen’s f^2^ was calculated for each model. Following Cohen’s conventions, f^2^ values of 0.02, 0.15 and 0.35 were considered small, medium, and large effects, respectively. The proportion of variance explained (R^2^) is reported.

Because the primary model was deliberately parsimonious, a sensitivity analysis was conducted in which the OLS models were re-fitted with additional covariates: age (continuous), marital status, BMI category, work schedule (shift vs. routine), chronic physical illness, chronic psychological illness, smoking status, weekly physical activity frequency, current diet status and self-rated health.

Descriptive, bivariate, and multivariable analyses were conducted using data from all 601 participants. Because the included variables were complete for all participants, no observations were excluded from the regression analyses. Results are presented as beta coefficients (β) with 95% confidence intervals (CIs). A two-sided *p* value of less than 0.05 was considered statistically significant.

### 2.11. Ethical Considerations

This study was approved by the Institutional Review Board of the Jazan Health Cluster (IRB No. 25176; 22 December 2025). All data were anonymized prior to analysis, and access to sensitive information was restricted to authorized personnel in accordance with ethical standards for human research.

Participation was voluntary. Electronic informed consent was obtained from all participants before questionnaire commission. No identifying information was collected, and data were stored securely with access restricted to the research team. Participants were informed that they could withdraw at any time without any consequences.

## 3. Results

### 3.1. Participant Characteristics

A total of 601 healthcare workers were included in the study. Most participants were male (63.7%), Saudi nationals (87.2%), and married (70.6%). Nearly half held a bachelor’s degree (47.1%), while 26.6% held a diploma and 19.0% had a board or doctoral qualification.

By profession, 43.1% were physicians/dentists, 32.0% were nurses, and 25.0% were allied health professionals. Participants were distributed across hospitals (49.4%) and primary healthcare centers (47.3%), and most reported a routine work schedule (75.2%) (Table 1).

Regarding health and lifestyle characteristics, 38.9% had normal BMI, 37.1% were overweight, and 19.8% were obese or extremely obese. Chronic physical illness was reported by 18.8%, and chronic psychological illness by 5.5%. Most participants were never-smokers (69.9%). Approximately one-third reported no weekly physical activity of at least 30 min per day (31.3%), whereas 13.2% reported physical activity on at least 5 days per week. Nearly one-quarter were currently following a diet (24.5%). Self-rated health was favorable, with most participants reporting very good or excellent health (74.7%). Almost all participants reported advising patients or family members about healthy lifestyle modification (97.5%) (Table 1).

### 3.2. CADA Capability

Because no validated cut-points or normative values are available for the CADA instrument, scores are interpreted descriptively relative to the 1–5 scale midpoint (3.0). In this study, mean scores within roughly ±0.5 points of the midpoint are described as reflecting an intermediate (“moderate”) level of perceived capability; this is a descriptive convention rather than a validated category. Comparisons with a previously published Saudi sample (Alenzi et al. [23]; mean Total CADA 3.28 ± 0.48) are reported. The mean Total CADA score was 3.28 ± 0.80. Overall, diet capability was higher than physical activity capability, with mean Diet CADA and Physical Activity CADA scores of 3.45 ± 0.85 and 3.11 ± 0.85, respectively (Table 2).

Among the diet-related domains, diet opportunity (3.79 ± 1.16) and diet knowledge (3.62 ± 1.19) had the highest mean scores, whereas diet time (3.13 ± 1.12) and diet barriers (3.17 ± 1.06) were lower. For physical activity, neighborhood opportunity (3.26 ± 1.14) and convenience (3.08 ± 1.24) showed moderate mean scores, while physical activity barriers had the lowest score (2.76 ± 1.00). Support-related domains were moderate for family support (3.19 ± 1.09) and nonfamily support (3.01 ± 1.12), whereas husband/wife support was markedly lower (1.25 ± 1.14) (Table 2).

#### Internal Consistency

Cronbach’s alpha for the full 34-item CADA scale (with negatively worded items reverse-coded) was α = 0.84, indicating good internal consistency in this healthcare-worker sample. Domain-level alphas were as follows: diet opportunity (5 items) α = 0.93; diet barriers (3 items) α = 0.80; diet knowledge (3 items) α = 0.91; diet time (3 items) α = 0.85; physical activity convenience (2 items) α = 0.78; physical activity neighborhood opportunity (5 items) α = 0.89; physical activity barriers (2 items) α = 0.53; physical activity time (2 items) α = 0.46; family support (3 items) α = 0.85; husband/wife support (3 items) α = 0.87; and nonfamily support (3 items) α = 0.88. The lower alpha values for the 2-item physical activity barriers and physical activity time sub-domains are typical of very short scales and reflect the limited number of items rather than poor content coherence. Corrected item-total correlations within each domain ranged from 0.46 to 0.87, and no item showed a corrected item-total correlation below 0.30. These results support the use of diet and physical activity composite scores in this sample. The internal consistency of the husband/wife support domain itself is acceptable (α = 0.87), but its interpretability remains limited by its low mean score and its applicability to the 27.1% of unmarried participants.

### 3.3. Bivariate Associations with Capability

In bivariate analyses, sex was associated with capability, with males showing higher mean Total CADA (3.34 ± 0.79 vs. 3.18 ± 0.80; *p* = 0.020) and Physical Activity CADA (3.19 ± 0.83 vs. 2.97 ± 0.86; *p* = 0.002) scores than females, whereas no significant sex difference was observed for Diet CADA (3.48 ± 0.85 vs. 3.39 ± 0.85; *p* = 0.217) (Table 3).

Education level and profession were significantly associated with Total, Diet, and Physical Activity CADA scores (all *p* < 0.001). In contrast, nationality and marital status were not significantly associated with Total or subscale CADA scores. Likewise, BMI category, chronic physical illness, chronic psychological illness, physical activity frequency, and current diet status were not significantly associated with capability scores (all *p* > 0.05). Smoking status showed a borderline association with Physical Activity CADA (*p* = 0.0511), but not with Total or Diet CADA scores (Table 3).

### 3.4. Multivariable Regression (Independent Predictors)

In multivariable ordinary least squares regression models adjusted for sex, education level, profession, and workplace, several independent associations remained statistically significant (Table 4).

Female sex was independently associated with lower Physical Activity CADA (β = −0.16; 95% CI −0.32 to −0.01; *p* = 0.042), whereas no significant adjusted association was found with Total or Diet CADA (*p* = 0.374 and *p* = 0.714, respectively). Compared with diploma holders, those with a bachelor’s degree had higher Total (β = 0.20; 95% CI 0.02 to 0.38; *p* = 0.026) and Diet (β = 0.21; 95% CI 0.03 to 0.40; *p* = 0.027) CADA scores, and those with board or doctoral qualifications had higher Total (β = 0.33; 95% CI 0.07 to 0.58; *p* = 0.013) and Physical Activity (β = 0.39; 95% CI 0.12 to 0.67; *p* = 0.005) CADA scores. Nurses and allied health professionals had lower adjusted Diet CADA scores than physicians/dentists (β = −0.29; 95% CI −0.50 to −0.08; *p* = 0.006 and β = −0.23; 95% CI −0.44 to −0.02; *p* = 0.031, respectively). Workplace setting was also independently associated with capability. Compared with hospital-based participants, those in primary healthcare centers had lower Total (β = −0.19; 95% CI −0.32 to −0.05; *p* = 0.007), Diet (β = −0.17; 95% CI −0.31 to −0.02; *p* = 0.024) and Physical Activity (β = −0.21; 95% CI −0.35 to −0.06; *p* = 0.006) CADA scores (Table 4).

Regression diagnostics indicated mild heteroskedasticity in the three primary models (Breusch–Pagan *p* = 0.006 for Total CADA, *p* = 0.007 for Diet CADA and *p* = 0.047 for Physical Activity CADA); robust (Huber–White) standard errors were therefore computed as a sensitivity check, and the statistical significance of all reported associations was preserved. Variance inflation factors for the predictors in the primary models were all below 2, indicating no problematic multicollinearity; the mean VIF was 3.13 in the fully adjusted sensitivity model (Supplementary Table S2), where higher VIFs were observed only for the individual job-title and detailed-workplace dummies that have many categories. The ordinal-logistic sensitivity analysis on the highest-loading items of each composite yielded results consistent with the OLS findings.

In the fully adjusted sensitivity model (N = 452 after listwise exclusion of records with missing work-schedule data), the direction of the principal associations, female sex with lower Total and Physical Activity CADA, and higher education with higher CADA scores, was preserved, although the standard errors widened and several previously borderline associations lost statistical significance. Specifically, the adjusted associations of female sex with Total CADA (β = −0.21; 95% CI −0.42 to −0.01; *p* = 0.039) and with Physical Activity CADA (β = −0.29; 95% CI −0.51 to −0.08; *p* = 0.007) became larger after additional adjustment, while age emerged as an independent negative predictor of Total CADA (β = −0.018 per year; *p* = 0.023) and Physical Activity CADA (β = −0.021 per year; *p* = 0.010). Normal, overweight, and obese BMI categories (relative to underweight) and chronic physical illness were positively associated with one or more CADA outcomes.

The PHC vs. hospital coefficient was attenuated and lost statistical significance (Total CADA β = −0.26; 95% CI −0.69 to 0.17; *p* = 0.231), reflecting both the reduction in N and the conditioning on additional variables that may lie on the causal pathway between workplace and perceived capability. Variance explained increased from R^2^ = 0.082 to R^2^ = 0.154 (Total CADA), from 0.078 to 0.144 (Diet CADA), and from 0.076 to 0.158 (Physical Activity CADA); however, the adjusted R^2^ changed only marginally (e.g., from 0.070 to 0.067 for Total CADA), indicating that most of the added predictors contributed limited unique explanatory power.

#### Sensitivity Analysis Regarding the Husband/Wife Support Domain

On reviewing the scoring code, the published Total CADA score in this manuscript (mean 3.28 ± 0.80) was computed as the mean of the diet-related items and physical-activity-related items only; the three support domains (family, husband/wife and nonfamily support) were not included in the published Total CADA. The very low husband/wife support mean (1.25 ± 1.14) therefore did not contribute to, or skew, the published Total CADA, Diet CADA, or Physical Activity CADA scores. To verify robustness, an alternative Total CADA’ score was nevertheless computed by adding all three support domains (mean of diet, physical activity and three support buckets); this gave Total CADA’ = 2.81 ± 0.68, which is lower than the published score precisely because of the very low spouse component. In a multivariable OLS model on Total CADA’ adjusted for the same covariates (sex, education, profession, workplace), the principal associations were preserved in direction and statistical significance: workplace setting (PHC vs hospital) remained significant (β = −0.12; 95% CI −0.24 to −0.001; *p* = 0.048), confirming that the substantive workforce-level findings are not driven by the spouse score in either direction. Full results of the sensitivity analysis are reported in Supplementary Table S3.

## 4. Discussion

The present study assessed perceived capability for healthy diet and physical activity among healthcare workers in the Jazan region, Saudi Arabia, using the CADA instrument. Because CADA was originally developed for the general population, our findings should be interpreted as reflecting how HCWs perceive their ability and opportunity to engage in these behaviors, rather than their actual dietary intake or physical-activity levels; no causal or behavioral inferences are drawn from these cross-sectional, self-reported data. The sample included HCWs of both sexes, a range of educational levels and professional categories, and both hospital- and PHC-based settings. Overall, perceived capability was intermediate (mean Total CADA 3.28 ± 0.80; scale midpoint 3.0), very similar to the previously reported Saudi population mean (3.28 ± 0.48; ref. [23]) but with greater variability, suggesting heterogeneity within the healthcare workforce. Perceived diet capability (3.45 ± 0.85) was higher than perceived physical activity capability (3.11 ± 0.85), consistent with regional reports of barriers to physical activity participation [28,29,30].

Because the CADA instrument captures self-perceived capability, the findings of this study should be interpreted as reflecting how healthcare workers perceive their ability and opportunity to engage in a healthy diet and physical activity, rather than their actual dietary intake or physical activity levels. Throughout the Discussion, the terms “diet capability” and “physical activity capability” therefore refer to perceived rather than measured behavior. Given the cross-sectional design and the absence of validated behavioral measures, no causal or behavioral inferences are drawn from these data.

Some determinants identified in this study, particularly education and gender, remain relevant in understanding perceived capability among healthcare workers. However, in our HCW sample, the gender effect was concentrated in physical activity capability, whereas education showed consistent positive associations with perceived capability. This is consistent with findings from regional studies indicating that both individual-level characteristics and occupational context contribute to variations in health-related patterns reported in previous studies among healthcare workers [28], although in the present study these findings reflect perceived capability rather than actual behavior.

Our findings suggest that workplace setting may be particularly important in HCWs, with PHC employment associated with lower Total, Diet, and Physical Activity CADA scores. Regional evidence further supports the role of workplace environment, with organizational structure and job demands influencing the perceived capability of healthcare workers to engage in healthy behaviors [31]. Occupation was also relevant, particularly for diet capability, with nurses and allied health professionals showing lower scores than physicians. Taken together, these findings may indicate that although some determinants overlap with population-based evidence, the pattern among HCWs may be more influenced by work environment and role-related factors.

Education showed a consistent positive association with perceived capability. Participants with higher educational attainment tended to have higher Total and Diet CADA scores, and stronger physical activity capability was also observed among those with board or doctoral qualifications. This pattern may reflect better health literacy, greater awareness of healthy lifestyle practices, and better access to supportive environments or resources among more highly educated HCWs. Similar associations have been reported in regional studies, where higher educational attainment has been linked to more favorable health-related patterns and greater awareness of healthy lifestyle practices [28]. Education may also be related to differences in job autonomy, schedule flexibility, and opportunities for engaging in healthy lifestyle practices, which may influence perceived capability rather than actual behavior. Although our study did not directly assess income or housing, the observed educational gradient suggests that structural and contextual factors may play an important role in shaping perceived capability among HCWs in Jazan [32,33].

Compared with hospital-based HCWs, those working in PHCs had lower Total, Diet and Physical Activity CADA scores. Although workplace factors such as workload, staffing, access to breaks, food availability during shifts and opportunities for movement during the workday have been proposed as potential explanations for differences of this kind, none of these factors was measured in the present study. Therefore, the observed PHC–hospital difference should be regarded as a hypothesis-generating finding that motivates future studies designed to measure these workplace exposures directly.

While statistically significant, the adjusted differences between hospital-based and PHC-based healthcare workers (β ranging from −0.17 to −0.21 on the 1–5 CADA scale; standardized |β*| around 0.11–0.15) correspond to roughly one-quarter of a standard deviation on the outcome and to small standardized effect sizes (Cohen’s f^2^ for the full models = 0.08–0.09; small by Cohen’s conventions). Given the sample size (N = 601), small mean differences can reach statistical significance without indicating a public-health-relevant gap. Therefore, these findings should be regarded as signals for further investigation rather than as evidence of substantive disparities in perceived capability.

Instead, gender differences in our study were more evident in relation to physical activity capability. This pattern may suggest that gender-related differences among HCWs are more evident in physical activity capability than in dietary capability. Such differences could be related to perceived time availability, competing responsibilities, or access to opportunities for exercise; however, these factors were not directly measured in this study. This interpretation is consistent with qualitative evidence suggesting that contextual and environmental factors may influence women’s physical activity-related experiences in the region [34].

In our study, BMI category was not significantly associated with Total, Physical Activity, or Diet CADA. Similarly, diagnosed chronic physical and psychological illnesses were not significantly associated with perceived capability. These findings may suggest that, among HCWs, perceived capability is less strongly differentiated by health status than in more heterogeneous community samples. These findings should be interpreted with caution: because CADA assesses perceived opportunity and constraint rather than measured intake or activity, the absence of an association with BMI or chronic illness does not imply absence of an association with actual behavior. It is also possible that the CADA instrument captures perceived opportunity and constraint rather than current clinical status or actual behavior [27].

Physical activity barriers had the lowest mean score among the assessed domains. The CADA items in this domain refer to perceived obstacles such as fatigue, time and competing responsibilities; we did not measure objective workload, working hours or sleep quality in this study, and the consistency of our pattern with regional reports of high workload and fatigue among healthcare workers should be regarded as descriptive rather than confirmatory. Recent evidence among healthcare workers similarly identifies perceived workload, time pressure, and fatigue as dominant barriers to physical activity participation [35]. Likewise, diet time and diet barriers scored lower than diet opportunity and diet knowledge, which may indicate that practical constraints are more limiting than awareness alone. This suggests that knowledge alone may be insufficient to improve perceived capability without supportive environmental and structural conditions.

Comparison with previous studies. Our overall Total CADA mean (3.28 ± 0.80) is nearly identical to the Saudi population-based estimate reported by Alenzi et al. [23] (3.28 ± 0.48), although our HCW sample showed greater variability, consistent with the heterogeneity introduced by occupational and workplace-related factors that are absent from general-population samples. Our finding that perceived physical activity capability is lower than perceived diet capability is in line with regional reports among healthcare workers in Qatar, the MENA region and India, where workload, fatigue and time pressure consistently emerge as the dominant barriers to physical activity [28,29,30,35]. The lower perceived capability observed among PHC-based participants is also broadly compatible with regional evidence of high workload and burnout in Jazan-based hospitals and primary care [24,25,26]. In contrast to studies relying on self-reported behavioral endpoints, the present study focuses on capability, a complementary construct that may explain why knowledge-based interventions alone often fail to change healthcare-worker behavior in this region.

Family and nonfamily support scores were moderate, whereas husband/wife support was markedly lower (1.25 ± 1.14). Three considerations should be acknowledged before interpreting this finding. First, items 29–31 are framed in a way that may have limited applicability to the 27.1% of unmarried participants, who selected the lowest response option (or the “not applicable” option, which was recoded as 0); this is consistent with a floor effect rather than uniformly poor spousal support. Second, the corrected item-total correlations and Cronbach’s alpha for this domain (α = 0.87) indicate that the items themselves are internally coherent, but that coherence does not imply applicability across the whole workforce. Third, and importantly, the published Total CADA, Diet CADA and Physical Activity CADA scores were computed without the husband/wife support items; therefore, the very low spouse score did not affect the principal analyses or the regression findings. Future studies using the CADA in mixed-marital-status workforce samples should consider either restricting the husband/wife support items to married participants or replacing them with a single “partner support” index that manages marital status more flexibly.

Overall, improving perceived capability for healthy lifestyle practices among HCWs may require capability-oriented workplace strategies rather than education alone. Interventions targeting perceived barriers to physical activity, access to healthy food options, and time pressure during the workday may help strengthen perceived capability. Given the consistently lower perceived capability observed among HCWs working in PHCs, workplace wellness initiatives in these settings may be especially important. Additional attention to groups with lower perceived capability, including female HCWs in relation to physical activity and non-physician staff in relation to diet, may also support more equitable capability across HCWs in workplace health promotion contexts.

Because the workplace and lifestyle factors invoked above (workload, staffing, food availability, break time, access to exercise facilities, working hours, household income) were not measured in the present study, these interpretations should be regarded as hypotheses for future workplace research rather than as conclusions of the present analysis. Because the present study is cross-sectional and based entirely on self-reported data, all observed associations should be interpreted as descriptive correlations rather than as evidence of a causal influence of occupational or workplace factors on perceived capability.

This study has several limitations. First, the use of self-reported data may have introduced both recall bias and social-desirability bias, particularly for weight, height (and therefore BMI), smoking, weekly physical activity frequency, current diet status and self-rated health, all of which were measured using single self-report items rather than validated instruments. Healthcare workers may have provided socially desirable responses to lifestyle items, which could have inflated some health-favorable responses. Second, the cross-sectional design precludes any causal inference between participant characteristics and CADA scores. Third, the use of stratified convenience sampling, combined with electronic recruitment through workplace channels and WhatsApp, is likely to have favored participation by HCWs who were more accessible, more digitally engaged or more interested in health-related topics; this is expected to bias the mean CADA scores upwards relative to the true Cluster average, and the observed associations may also be attenuated if non-respondents differ systematically from respondents.

Fourth, no formal census of the Jazan Health Cluster workforce is publicly available, so the response rate could only be estimated. Fifth, the very low husband/wife support score should be interpreted considering the applicability issues. Sixth, several workplace and lifestyle variables identified by reviewers as potentially relevant, including workload intensity, weekly working hours, burnout, sleep quality, perceived stress, commuting, household income, and access to exercise facilities and food availability during shifts, were not measured, and the associations interpreted in relation to these factors should therefore be regarded as hypothesis-generating. Generalizability beyond governmental HCWs in the Jazan region is limited.

In summary, HCWs in Jazan demonstrated moderate perceived capability to adopt healthy lifestyle behaviors, with higher perceived capability for diet compared with physical activity. Educational level and workplace setting were important correlates of perceived capability, while gender differences were more evident in physical activity capability. These findings may help inform the development of workplace-based strategies aimed at reducing barriers to healthy behaviors among HCWs, particularly in primary healthcare settings.

## 5. Conclusions

Healthcare workers in the Jazan health cluster demonstrated moderate perceived capability to adopt healthy diet and physical activity behaviors, with stronger perceived capability for diet than for physical activity. Educational level, profession, workplace setting, and female sex (in relation to perceived physical activity capability) were the variables most associated with perceived capability. Lower perceived capability among healthcare workers in primary healthcare centers suggests that workplace context may influence how staff perceive their opportunities and constraints related to healthy lifestyle behaviors, although causal inference is not possible from the present cross-sectional, self-reported data. These findings indicate that workplace-based interventions addressing perceived barriers, particularly those related to physical activity, time pressure, and access to supportive environments, may need evaluation. Future longitudinal studies combining the CADA instrument with validated behavioral measures (e.g., IPAQ for physical activity and a food-frequency questionnaire for diet) are needed to clarify how perceived capability relates to actual behaviors and health outcomes among healthcare workers.

## Figures and Tables

**Table 1:** Participant characteristics of the Study Population.

Variable	Category	n	%
Sex	Male	383	63.73
	Female	218	36.27
Nationality	Saudi	524	87.19
	Non-Saudi	77	12.81
Marital Status	Single	163	27.12
	Married	424	70.55
	Divorced	14	2.33
Education Level	Diploma	160	26.62
	Bachelor’s degree	283	47.09
	Master’s degree	44	7.32
	Board/doctoral qualification	114	18.97
Profession	Physicians & dentists	259	43.09
	Nurses	192	31.95
	Allied health professionals *	150	24.96
Workplace	Hospital	297	49.42
	Primary healthcare center	284	47.25
	Other **	20	3.33
Work Schedule	Shifting schedule	149	24.79
	Routine schedule	452	75.21
BMI Category	Underweight	25	4.16
	Normal	234	38.94
	Overweight	223	37.10
	Obese	86	14.31
	Extremely obese	33	5.49
Chronic Physical Illness	No	488	81.20
	Yes	113	18.80
Chronic psychological illness	No	568	94.51
	Yes	33	5.49
Smoking Status	Never	420	69.88
	Passive	32	5.32
	Former smoker	54	8.99
	Current smoker	95	15.81
Physical Activity Frequency	0 days/week	188	31.28
	1–2 days/week	190	31.61
	3–4 days/week	144	23.96
	5–6 days/week	40	6.66
	7 days/week	39	6.49
Current diet status	No	454	75.54
	Yes	147	24.46
Advises patients on lifestyle	No	15	2.50
	Yes	586	97.50
Self-Rated Health	Poor	8	1.33
	Fair	24	3.99
	Good	120	19.97
	Very good	210	34.94
	Excellent	239	39.77

Notes: Data are presented as frequency and percentage. * Allied health professionals included assistant dentists, clinical nutritionists, health educators, laboratory specialists and technicians, paramedics, pharmacists, physiotherapists, public health specialists and technicians, radiology specialists and technicians, and respiratory therapists. ** Other workplaces included administrative and fieldwork settings.

**Table 2:** Descriptive statistics of Capability Assessment for Diet and Activity (CADA).

Variables	Mean	Std. Deviation
1. Total CADA score	3.28	0.80
2. Diet CADA score	3.45	0.85
2.1 Diet opportunity (items 1, 4–7)	3.79	1.16
2.2 Diet barriers (items 14, 15, 17)	3.17	1.06
2.3 Diet knowledge (items 18–20)	3.62	1.19
2.4 Diet time (items 22–24)	3.13	1.12
3. Physical Activity CADA score	3.11	0.85
3.1 Physical activity convenience (items 2–3)	3.08	1.24
3.2 Neighborhood (items 8–12)	3.26	1.14
3.3 Physical activity barriers (items 13, 16)	2.76	1.00
3.4 Physical activity time (items 21, 25)	3.11	1.02
4. Family support (items 26–28)	3.19	1.09
5. Husband/wife support (items 29–31)	1.25	1.14
6. Nonfamily support (items 32–34)	3.01	1.12

Notes: Global item numbering (1–34) follows the questionnaire structure: convenience/cost (items 1–7), neighborhood opportunity (8–12), barriers (13–17), knowledge (18–20), time pressure (21–25), family support (26–28), husband/wife support (29–31), and nonfamily support (32–34). Higher scores indicate greater capability.

**Table 3:** Bivariate associations between participant characteristics and CADA scores.

Variables	Overall CADA Score		Overall Physical Activity Score		Overall Diet Score	
	X ± SD	*p*-Value	X ± SD	*p*-Value	X ± SD	*p*-Value
**Sociodemographic data**						
Gender						
Male	3.34 ± 0.79	**0.0196**	3.19 ± 0.83	**0.0016**	3.48 ± 0.85	0.2167
Female	3.18 ± 0.80		2.97 ± 0.86		3.39 ± 0.85	
Nationality						
Saudi	3.28 ± 0.80	0.7069	3.12 ± 0.84	0.4719	3.45 ± 0.86	0.9909
Non-Saudi	3.25 ± 0.77		3.04 ± 0.87		3.45 ± 0.81	
Marital status						
Single	3.34 ± 0.73		3.18 ± 0.79		3.51 ± 0.79	
Married	3.25 ± 0.82	0.3351	3.08 ± 0.87	0.3857	3.42 ± 0.88	0.3724
Divorced	3.43 ± 0.56		3.23 ± 0.63		3.62 ± 0.66	
Education level						
Diploma	3.00 ± 0.93		2.85 ± 0.94		3.14 ± 0.99	
Bachelor	3.34 ± 0.76		3.15 ± 0.82		3.53 ± 0.81	
Master	3.22 ± 0.68	**<0.001**	3.07 ± 0.78	**<0.001**	3.36 ± 0.70	**<0.001**
Board/Doctoral	3.55 ± 0.60		3.40 ± 0.68		3.71 ± 0.63	
Occupation						
Physicians/dentists	3.47 ± 0.64		3.28 ± 0.73		3.67 ± 0.68	
Nurse	3.10 ± 0.88	**<0.001**	2.95 ± 0.92	**<0.001**	3.25 ± 0.92	**<0.001**
Allied health professionals	3.17 ± 0.86		3.02 ± 0.88		3.32 ± 0.93	
**Health status**						
BMI category						
Underweight	3.03 ± 1.08		2.82 ± 1.06		3.23 ± 1.15	
Normal	3.31 ± 0.74		3.16 ± 0.80		3.46 ± 0.80	
Overweight	3.28 ± 0.80	0.511	3.09 ± 0.85	0.364	3.47 ± 0.85	0.713
Obese	3.29 ± 0.81		3.14 ± 0.81		3.43 ± 0.89	
Extremely obese	3.19 ± 0.89		3.01 ± 1.04		3.37 ± 0.85	
Chronic physical disease						
Yes	3.33 ± 0.78	0.4634	3.15 ± 0.86	0.5473	3.50 ± 0.81	0.4381
No	3.27 ± 0.80		3.10 ± 0.84		3.44 ± 0.86	
Chronic psychological disease						
Yes	3.38 ± 0.77	0.4496	3.22 ± 0.81	0.4396	3.54 ± 0.87	0.5170
No	3.27 ± 0.80		3.10 ± 0.85		3.44 ± 0.85	
**Lifestyle**						
Smoking status						
Never	3.25 ± 0.79		3.07 ± 0.85		3.44 ± 0.84	
Passive	3.48 ± 0.59		3.30 ± 0.67		3.66 ± 0.68	
Former smoker	3.15 ± 0.98	0.1096	3.02 ± 1.02	0.0511	3.28 ± 1.03	0.2033
Current smoker	3.40 ± 0.76		3.29 ± 0.76		3.51 ± 0.84	
Physical activity frequency						
0 days	3.26 ± 0.81		3.05 ± 0.87		3.47 ± 0.87	
1–2 days	3.28 ± 0.77		3.13 ± 0.83		3.44 ± 0.81	
3–4 days	3.27 ± 0.84	0.8616	3.11 ± 0.85	0.4195	3.43 ± 0.92	0.9894
5–6 days	3.27 ± 0.74		3.10 ± 0.79		3.43 ± 0.97	
7 days	3.41 ± 0.75		3.33 ± 0.81		3.49 ± 0.77	
Diet status						
Yes	3.26 ± 0.77	0.7096	3.04 ± 0.83	0.2564	3.47 ± 0.82	0.6659
No	3.29 ± 0.80		3.13 ± 0.85		3.44 ± 0.86	

Notes: Data are presented as mean ± SD. Independent-samples *t*-tests were used for dichotomous variables and one-way ANOVA for variables with more than two categories. Bold *p*-values indicate statistical significance at *p* < 0.05.

**Table 4:** Adjusted associations with CADA scores (multivariable linear regression).

Variables	Overall CADA Score		Overall Physical Activity Score		Overall Diet Score	
	β (95% CI)	*p*-Value	β (95% CI)	*p*-Value	β (95% CI)	*p*-Value
Gender						
Male **(Ref.)**						
Female	−0.07 (−0.22 to 0.08)	0.374	−0.16 (−0.32 to −0.01)	**0.042**	0.03 (−0.13 to 0.19)	0.714
Education level						
Diploma **(Ref.)**						
Bachelor	0.20 (0.02 to 0.38)	**0.026**	0.19 (0.00 to 0.38)	0.051	0.21 (0.03 to 0.40)	**0.027**
Master	0.08 (−0.19 to 0.35)	0.564	0.10 (−0.19 to 0.39)	0.497	0.06 (−0.23 to 0.35)	0.687
Board/Doctoral	0.33 (0.07 to 0.58)	**0.013**	0.39 (0.12 to 0.67)	**0.005**	0.26 (−0.02 to 0.53)	0.064
Occupation						
Physicians/dentist (Ref.)						
Nurses	−0.17 (−0.37 to 0.02)	0.080	−0.06 (−0.27 to 0.15)	0.587	−0.29 (−0.50 to −0.08)	**0.006**
Allied health professionals	−0.16 (−0.36 to 0.03)	0.101	−0.10 (−0.30 to 0.11)	0.361	−0.23 (−0.44 to −0.02)	**0.031**
Workplace						
Hospital **(Ref.)**						
PHC	−0.19 (−0.32 to −0.05)	**0.007**	−0.21 (−0.35 to −0.06)	**0.006**	−0.17 (−0.31 to −0.02)	**0.024**
Others	−0.20 (−0.56 to 0.15)	0.255	−0.25 (−0.63 to 0.13)	0.193	−0.16 (−0.54 to 0.22)	0.406

Notes: β = regression coefficient; CI = confidence interval; Ref. = reference category. Bold *p*-values indicate statistical significance at *p* < 0.05. Models were adjusted for sex, education level, profession, and workplace. Although several adjusted associations reached statistical significance, the magnitude of the standardized coefficients was modest (|β*| ≤ 0.16 across all models) and the variance explained was small: R^2^ = 0.082 (Adjusted R^2^ = 0.070) for Total CADA, R^2^ = 0.078 (Adjusted R^2^ = 0.065) for Diet CADA, and R^2^ = 0.076 (Adjusted R^2^ = 0.063) for Physical Activity CADA. Cohen’s f^2^ values for the three models were 0.089, 0.084 and 0.082, respectively—all corresponding to small effect sizes by Cohen’s conventions (f^2^ ≥ 0.02 small, ≥0.15 medium, ≥0.35 large).

## Data Availability

The datasets generated and/or analyzed during the current study are not publicly available due to privacy and ethical restrictions involving healthcare workers’ data but are available from the corresponding author on reasonable request.

## Supplementary Materials

The following supporting information can be downloaded at https://www.mdpi.com/article/10.3390/healthcare14111530/s1: Table S1: Capability Assessment for Diet and Activity (CADA) questionnaire; Table S2: Fully adjusted multivariable OLS regression models for Total CADA, Diet CADA and Physical Activity CADA (N = 452); Table S3: Sensitivity analysis of the published multivariable models when the husband/wife support domain is added to the CADA composite (N = 601).

## Abbreviations

The following abbreviations are used in this manuscript:

ANOVA	Analysis of variance
BMI	Body mass index
CADA	Capability Assessment for Diet and Activity
CI	Confidence interval
HCWs	Healthcare workers
IRB	Institutional Review Board
KSA	Kingdom of Saudi Arabia
NCDs	Non-communicable diseases
OLS	Ordinary least squares
PHC	Primary healthcare center
SD	Standard deviation
