# Supplementary material for: Capability Assessment for Diet and Activity (CADA) and Its Influencing Factors Among Healthcare Workers in the Jazan Region, Saudi Arabia, 2026: A Cross-Sectional Study
Source: Healthcare (Basel). 2026 Jun 1;14(11):1530. doi: 10.3390/healthcare14111530 (PMC13256563; doi:10.3390/healthcare14111530)
Supplement: Supplementary file 1 [file healthcare-14-01530-s001.zip › Supplementary_Tables_S2_healthcare-4309957.pdf]

Supplementary Table S2. Fully adjusted multivariable OLS regression models for Total CADA, Diet CADA and Physical Activity CADA (N = 452)

	Total CADA			Diet CADA			Physical Activity CADA		
**Predictor**	**β**	**95% CI**	***p***	**β**	**95% CI**	***p***	**β**	**95% CI**	***p***
Sociodemographic									
Female (vs. Male)	**-0.21**	(-0.42, -0.01)	**0.039**	-0.13	(-0.35, 0.08)	0.234	**-0.29**	(-0.51, -0.08)	**0.007**
Non-Saudi (vs. Saudi)	0.05	(-0.28, 0.39)	0.751	0.02	(-0.34, 0.38)	0.917	0.09	(-0.27, 0.45)	0.622
Age (years)	**-0.02**	(-0.03, -0.00)	**0.023**	-0.01	(-0.03, 0.00)	0.088	**-0.02**	(-0.04, -0.01)	**0.010**
Marital status (vs. Single)									
Married	0.03	(-0.17, 0.23)	0.754	0.01	(-0.20, 0.22)	0.920	0.05	(-0.16, 0.26)	0.626
Divorced	0.04	(-0.46, 0.55)	0.873	0.12	(-0.42, 0.66)	0.667	-0.04	(-0.57, 0.50)	0.893
Education (vs. Diploma)									
Bachelor	0.08	(-0.15, 0.32)	0.490	0.16	(-0.10, 0.41)	0.221	0.01	(-0.24, 0.26)	0.950
Master	-0.05	(-0.40, 0.31)	0.793	-0.07	(-0.45, 0.31)	0.713	-0.02	(-0.40, 0.35)	0.902
Board/Doctoral	0.12	(-0.20, 0.44)	0.461	0.13	(-0.21, 0.47)	0.466	0.11	(-0.23, 0.45)	0.516
Profession (vs. Assistant dentist)*									
Clinical nutrition	-0.16	(-0.85, 0.53)	0.653	-0.44	(-1.18, 0.31)	0.249	0.12	(-0.61, 0.85)	0.748
Dentist	-0.36	(-1.06, 0.34)	0.319	-0.43	(-1.18, 0.32)	0.260	-0.28	(-1.02, 0.46)	0.461
Health educator	-0.70	(-1.48, 0.07)	0.076	**-0.88**	(-1.71, -0.04)	**0.040**	-0.53	(-1.36, 0.30)	0.208
Laboratory	0.13	(-0.46, 0.73)	0.657	0.06	(-0.58, 0.70)	0.850	0.21	(-0.42, 0.84)	0.518
Nurse	-0.22	(-0.74, 0.29)	0.395	-0.37	(-0.93, 0.18)	0.187	-0.07	(-0.62, 0.47)	0.790
Paramedic	-0.64	(-1.70, 0.43)	0.242	-0.76	(-1.91, 0.39)	0.194	-0.51	(-1.65, 0.62)	0.373
Pharmacist	-0.37	(-1.08, 0.34)	0.301	-0.46	(-1.22, 0.30)	0.239	-0.29	(-1.04, 0.46)	0.450
Physiotherapy	-0.63	(-1.68, 0.42)	0.241	-0.99	(-2.12, 0.14)	0.085	-0.26	(-1.38, 0.85)	0.642
Public health	-0.25	(-0.80, 0.31)	0.386	-0.32	(-0.92, 0.28)	0.292	-0.17	(-0.76, 0.42)	0.571
Radiology	-0.25	(-0.88, 0.38)	0.442	-0.30	(-0.98, 0.38)	0.383	-0.19	(-0.86, 0.48)	0.573
Respiratory therapist	0.47	(-1.14, 2.09)	0.566	0.26	(-1.47, 1.99)	0.767	0.68	(-1.03, 2.40)	0.435
Physician	-0.09	(-0.64, 0.47)	0.756	-0.17	(-0.76, 0.43)	0.585	-0.01	(-0.60, 0.58)	0.974

	Total CADA			Diet CADA			Physical Activity CADA		
**Predictor**	**β**	**95% CI**	***p***	**β**	**95% CI**	***p***	**β**	**95% CI**	***p***
Workplace (vs. Administrative)									
Fieldwork	1.34	(-0.34, 3.03)	0.118	1.36	(-0.45, 3.17)	0.141	1.33	(-0.46, 3.12)	0.145
Hospital	-0.09	(-0.52, 0.34)	0.697	-0.16	(-0.63, 0.30)	0.488	-0.01	(-0.47, 0.45)	0.975
Hospital + Administrative	-0.16	(-1.21, 0.88)	0.762	-0.30	(-1.42, 0.82)	0.600	-0.02	(-1.13, 1.09)	0.969
PHC	-0.26	(-0.69, 0.17)	0.231	-0.33	(-0.79, 0.13)	0.163	-0.20	(-0.65, 0.26)	0.399
PHC + Administrative	0.27	(-0.49, 1.02)	0.489	0.44	(-0.37, 1.25)	0.287	0.09	(-0.71, 0.89)	0.822
PHC + Fieldwork	0.87	(-0.31, 2.05)	0.148	0.59	(-0.68, 1.86)	0.362	1.15	(-0.10, 2.41)	0.071
PHC + Hospital	0.00	(-0.70, 0.71)	0.991	-0.10	(-0.86, 0.66)	0.800	0.11	(-0.64, 0.85)	0.782
Health status									
Normal BMI (vs. Underweight)	0.43	(-0.01, 0.87)	0.055	**0.50**	(0.03, 0.98)	**0.037**	0.36	(-0.11, 0.83)	0.134
Overweight (vs. Underweight)	**0.45**	(0.01, 0.89)	**0.046**	**0.55**	(0.08, 1.03)	**0.022**	0.34	(-0.13, 0.81)	0.153
Obese (vs. Underweight)	**0.50**	(0.03, 0.97)	**0.037**	**0.58**	(0.08, 1.09)	**0.024**	0.42	(-0.08, 0.92)	0.099
Extremely obese (vs. Underweight)	0.23	(-0.32, 0.78)	0.408	0.32	(-0.28, 0.91)	0.294	0.15	(-0.44, 0.73)	0.619
Chronic physical illness (vs. No)	0.19	(-0.02, 0.39)	0.072	0.13	(-0.09, 0.35)	0.236	**0.24**	(0.03, 0.46)	**0.028**
Chronic mental illness (vs. No)	-0.01	(-0.38, 0.36)	0.949	-0.07	(-0.46, 0.33)	0.742	0.04	(-0.35, 0.43)	0.832
Lifestyle									
Passive smoking (vs. Never)	-0.03	(-0.39, 0.32)	0.860	0.00	(-0.38, 0.38)	0.993	-0.06	(-0.44, 0.32)	0.745
Former smoker (vs. Never)	-0.02	(-0.32, 0.27)	0.875	-0.12	(-0.44, 0.20)	0.460	0.07	(-0.24, 0.39)	0.651
Current smoker (vs. Never)	0.03	(-0.20, 0.26)	0.775	-0.05	(-0.30, 0.19)	0.670	0.12	(-0.12, 0.36)	0.332
Physical activity frequency (vs. 0 days)									
1–2 days/week	-0.01	(-0.20, 0.18)	0.935	-0.11	(-0.31, 0.10)	0.314	0.09	(-0.11, 0.29)	0.386
3–4 days/week	0.00	(-0.21, 0.22)	0.986	-0.06	(-0.29, 0.17)	0.599	0.07	(-0.16, 0.30)	0.571
5–6 days/week	0.15	(-0.17, 0.47)	0.349	0.03	(-0.32, 0.37)	0.878	0.28	(-0.06, 0.62)	0.108
7 days/week	0.30	(-0.05, 0.66)	0.088	0.13	(-0.25, 0.51)	0.502	**0.48**	(0.11, 0.85)	**0.011**
Currently on a diet (vs. No)	0.06	(-0.13, 0.24)	0.535	0.14	(-0.05, 0.34)	0.156	-0.03	(-0.22, 0.17)	0.789
Self-rated health (1–5 scale, continuous)	0.04	(-0.06, 0.13)	0.450	0.00	(-0.10, 0.10)	0.936	0.07	(-0.03, 0.17)	0.180

Notes. *β = unstandardised regression coefficient on the 1–5 CADA scale; CI = confidence interval; p from a two-sided test; bold = p < 0.05. Coefficients are taken directly from the Stata 15 OLS output (regress command with i. factor-variable expansion for categorical predictors and c. for continuous predictors). The work-schedule variable (jobshift_num) was omitted because of collinearity. Reference categories: see "Purpose" above.*

****Note on the profession reference category:*** *Because the full 12-category job_num variable is used in this fully adjusted model, the reference category becomes the first level (Assistant dentist; n = 11), which is different from the collapsed 3-category prof3 variable used in the parsimonious published model (where Physicians/Dentists is the reference; n = 259). Differences between professions in this table should therefore be interpreted with the small Assistant-dentist reference cell in mind and with appropriate caution given the wide confidence intervals.*
