# Supplementary material for: Capability Assessment for Diet and Activity (CADA) and Its Influencing Factors Among Healthcare Workers in the Jazan Region, Saudi Arabia, 2026: A Cross-Sectional Study
Source: Healthcare (Basel). 2026 Jun 1;14(11):1530. doi: 10.3390/healthcare14111530 (PMC13256563; doi:10.3390/healthcare14111530)
Supplement: Supplementary file 1 [file healthcare-14-01530-s001.zip › Supplementary_Tables_S3_healthcare-4309957.pdf]

Supplementary Table S3. Sensitivity analysis of the published multivariable models when the husband/wife support domain is added to the CADA composite (N = 601)

Outcome: Total CADA (published: diet + physical activity items; sensitivity: diet + physical activity + husband/wife support items)						
Model fit	*Mean 3.28 ± 0.80 | R^2 = 0.0819 | Adj R^2 = 0.0695 | F = 6.60, p <0.001*			*Mean 2.61 ± 0.68 | R^2 = 0.0569 | Adj R^2 = 0.0442 | F = 4.47, p <0.001*		
	**Published model**			**Sensitivity: + husband/wife domain**		
**Predictor**	**β**	**95% CI**	***p***	**β**	**95% CI**	***p***
Female (vs. Male)	-0.07	(-0.22, 0.08)	0.374	-0.13	(-0.25, 0.00)	0.051
Education level (vs. Diploma)						
Bachelor	**0.20**	(0.02, 0.38)	**0.026**	-0.06	(-0.21, 0.09)	0.404
Master	0.08	(-0.19, 0.35)	0.564	-0.02	(-0.25, 0.21)	0.848
Board/Doctoral	**0.33**	(0.07, 0.58)	**0.013**	0.08	(-0.13, 0.30)	0.430
Profession (vs. Physicians/Dentists)						
Nurses	-0.17	(-0.37, 0.02)	0.080	**-0.19**	(-0.35, -0.03)	**0.023**
Allied health professionals	-0.16	(-0.36, 0.03)	0.101	-0.07	(-0.24, 0.09)	0.366
Workplace (vs. Hospital)						
Primary healthcare centre	**-0.19**	(-0.32, -0.05)	**0.007**	**-0.12**	(-0.24, 0.00)	**0.048**
Other (admin / fieldwork)	-0.20	(-0.56, 0.15)	0.255	-0.12	(-0.37, 0.14)	0.360

Outcome: Diet CADA (published: diet items; sensitivity: diet + husband/wife support items)						
Model fit	*Mean 3.45 ± 0.85 | R^2 = 0.0778 | Adj R^2 = 0.0653 | F = 6.24, p <0.001*			*Mean 2.36 ± 0.73 | R^2 = 0.0488 | Adj R^2 = 0.0360 | F = 3.80, p <0.001*		
	**Published model**			**Sensitivity: + husband/wife domain**		
**Predictor**	**β**	**95% CI**	***p***	**β**	**95% CI**	***p***
Female (vs. Male)	0.03	(-0.13, 0.19)	0.714	-0.11	(-0.25, 0.03)	0.127
Education level (vs. Diploma)						
Bachelor	**0.21**	(0.03, 0.40)	**0.027**	**-0.18**	(-0.34, -0.02)	**0.028**
Master	0.06	(-0.23, 0.35)	0.687	-0.07	(-0.33, 0.18)	0.565
Board/Doctoral	0.26	(-0.02, 0.53)	0.064	-0.05	(-0.28, 0.18)	0.647

Profession (vs. Physicians/Dentists)						
Nurses	**-0.29**	(-0.50, -0.08)	**0.006**	**-0.25**	(-0.42, -0.07)	**0.006**
Allied health professionals	**-0.23**	(-0.44, -0.02)	**0.031**	-0.04	(-0.21, 0.14)	0.671
Workplace (vs. Hospital)						
Primary healthcare centre	**-0.17**	(-0.31, -0.02)	**0.024**	-0.07	(-0.19, 0.06)	0.313
Other (admin / fieldwork)	-0.16	(-0.54, 0.22)	0.406	-0.12	(-0.40, 0.15)	0.378

Outcome: Physical Activity CADA (published: physical activity items; sensitivity: physical activity + husband/wife support items)						
Model fit	*Mean 3.11 ± 0.85 | R^2 = 0.0755 | Adj R^2 = 0.0630 | F = 6.04, p <0.001*			*Mean 2.18 ± 0.73 | R^2 = 0.0576 | Adj R^2 = 0.0449 | F = 4.52, p <0.001*		
	**Published model**			**Sensitivity: + husband/wife domain**		
**Predictor**	**β**	**95% CI**	***p***	**β**	**95% CI**	***p***
Female (vs. Male)	**-0.16**	(-0.32, -0.01)	**0.042**	**-0.18**	(-0.32, -0.04)	**0.010**
Education level (vs. Diploma)						
Bachelor	0.19	(0.00, 0.38)	0.051	**-0.22**	(-0.38, -0.05)	**0.009**
Master	0.10	(-0.19, 0.39)	0.497	-0.07	(-0.32, 0.18)	0.585
Board/Doctoral	**0.39**	(0.12, 0.67)	**0.005**	-0.01	(-0.24, 0.22)	0.915
Profession (vs. Physicians/Dentists)						
Nurses	-0.06	(-0.27, 0.15)	0.587	-0.13	(-0.31, 0.04)	0.133
Allied health professionals	-0.10	(-0.30, 0.11)	0.361	0.03	(-0.15, 0.20)	0.762
Workplace (vs. Hospital)						
Primary healthcare centre	**-0.21**	(-0.35, -0.06)	**0.006**	-0.08	(-0.21, 0.05)	0.209
Other (admin / fieldwork)	-0.25	(-0.63, 0.13)	0.193	-0.15	(-0.43, 0.13)	0.291

Notes. *β = unstandardised regression coefficient on the 1–5 CADA scale; CI = confidence interval; p from a two-sided test; bold = $p < 0.05$. Published-model coefficients are taken from the Stata output reproduced in Table 4 of the main manuscript (cada_total, cada_diet, cada_pa; N = 601). Sensitivity-model coefficients are from re-fitted OLS models on the same N = 601 sample, with the outcome redefined as the mean of the original composite and the three husband/wife items. All models adjust for sex, education, profession, and workplace.*
