# Supplementary material for: Capability Assessment for Diet and Activity (CADA) and Its Influencing Factors Among Healthcare Workers in the Jazan Region, Saudi Arabia, 2026: A Cross-Sectional Study
Source: Healthcare (Basel). 2026 Jun 1;14(11):1530. doi: 10.3390/healthcare14111530 (PMC13256563; doi:10.3390/healthcare14111530)
Supplement: Supplementary file 1 [file healthcare-14-01530-s001.zip › Table S1.pdf]

Capability Assessment for Diet and Activity (CADA) questionnaire

Data Collection Form:

Study Title: Capability Assessment for Diet and Activity (CADA) and Its Influencing Factors Among Healthcare Workers in the Jazan Region, Saudi Arabia, 2026: A Cross-Sectional Study

Instructions:

This questionnaire aims to assess factors influencing healthy dietary habits and physical activity. There are no right or wrong answers. Please read each question carefully and select the option that best reflects your situation. All responses are confidential and will be used for research purposes only.

Section 1: Sociodemographic Information

Age (years)	_______Years
Gender	□ Male<br>□ Female
Marital status	□ Single<br>□ Married<br>□ Divorced<br>□ Widowed
Nationality	□ Saudi<br>□ Non-Saudi
Highest education level	□ Diploma<br>□ Bachelor<br>□ Master degree<br>□ Board or Doctoral degree

Job title	□ physician<br>□ Dentist<br>□ Assist dentists<br>□ Nurse<br>□ Radiology<br>□ Physiotherapy<br>□ Laboratory<br>□ Respiratory therapist<br>□ Clinical nutrition<br>□ Pharmacist<br>□ Public health<br>□ Health educator
Place of Work	□ PHC<br>□ Hospital<br>□ Adminstrivie
Current Job	□ Routine schedule 8 AM to 4 PM<br>□ shifting schedule: morning, evening, night

<u>Section 2: Health Status</u>

Weight	______KG
Hight	______CM
Self-rated general health	□ Excellent<br>□ Very good<br>□ Good

	□ Fair<br>□ Poor
Diagnosed chronic illness (physical)	□ Yes<br>□ No
If previous question answer (Yes) What is the chronic disease: can choose more than one answer?	□ prediabetes<br>□ Type 1 Dm<br>□ Type 2 DM<br>□ HTN<br>□ Dyslipidemia<br>□ Metabolic syndrome<br>□ polycystic ovarian disease (women)
Diagnosed mental health condition	□ Yes<br>□ No
If previous question (Yes): What is specific mental illness diagnosed?	□ Anxiety<br>□ Depression<br>□ Stress<br>□ Burnout
Current smoking status	□ Never<br>□ Current<br>□ Passive
If previous question (Yes): What types of nicotine used?	□ Shisha<br>□ Cigarettes smoker<br>□ Pouch
Physically active ≥30 min/day	□ 0 days/week<br>□ 1–2<br>□ 3–4

	□ 5–6<br>□ 7
Following a special diet	□ Yes<br>□ No
If previous question answers (Yes) what specific diet?: can choose more than one answer	□ low carb diet<br>□ low caloric<br>□ eat fruit and vegetables<br>□ low fat
Are you advice your patients and family about healthy life?	□ Yes<br>□ NO

<u>Section 3: CADA Items</u>

Please use the following response scale:

1 = Almost never 2 = Rarely 3 = Sometimes 4 = Often 5 = Almost always

A- Convenience, cost

Item	Statement	1	2	3	4	5
1	Easy to get to store for food shopping	□	□	□	□	□
2	Nearby places for outdoor physical activity	□	□	□	□	□
3	Places open when I want to do indoor physical activity	□	□	□	□	□
4	Fresh fruits and vegetables available where I shop for food	□	□	□	□	□
5	I can afford to buy fresh fruits and vegetables	□	□	□	□	□
6	I can afford to buy fish or lean meat	□	□	□	□	□

7	Fruits and vegetables where I shop are high quality	◻	◻	◻	◻	◻

B- Neighborhood opportunity

Item	Statement	1	2	3	4	5
8	In my neighborhood it is easy to walk places	◻	◻	◻	◻	◻
9	Places where I can be active without needing to pay	◻	◻	◻	◻	◻
10	I often see other people walking in my neighborhood	◻	◻	◻	◻	◻
11	People generally feel safe in my neighborhood	◻	◻	◻	◻	◻
12	My neighborhood is well lighted for evening activities	◻	◻	◻	◻	◻

C- Barriers

Item	Statement	1	2	3	4	5
13	I am too tired to be physically active	◻	◻	◻	◻	◻
14	Illness gets in the way of cooking own meals	◻	◻	◻	◻	◻
15	I am too tired to cook my own meals	◻	◻	◻	◻	◻
16	Feeling depressed keeps me from being active	◻	◻	◻	◻	◻
17	Feeling depressed keeps me from shopping for food	◻	◻	◻	◻	◻

D- Knowledge

Item	Statement	1	2	3	4	5
18	I know how to eat healthy foods	□	□	□	□	□
19	When I eat at a restaurant, I know how to choose a healthy meal	□	□	□	□	□
20	I know where in my neighborhood to shop for healthy foods	□	□	□	□	□

E- Time pressure

Item	Statement	1	2	3	4	5
21	Taking care of my family gives little time to be physically active	□	□	□	□	□
22	Taking care of my family gives me little time to cook meals	□	□	□	□	□
23	My schedule gives me little time to cook my own meals	□	□	□	□	□
24	My schedule gives me little time to go food shopping	□	□	□	□	□
25	I have time to be physically active on most days	□	□	□	□	□

F- Family support

Item	Statement	1	2	3	4	5
26	There are people I live with who eat healthy foods	□	□	□	□	□
27	There are people I live with who are physically active	□	□	□	□	□

28	Family allows me to eat recommended foods	□	□	□	□	□

G- Spouse/partner

Item	Statement	1	2	3	4	5
29	Spouse or partner complains when I serve a healthy meal	□	□	□	□	□
30	Spouse or partner doesn't allow me to be physically active	□	□	□	□	□
31	When I plan my day, I have to think about my partner's jealousy	□	□	□	□	□

H- Nonfamily support

Item	Statement	1	2	3	4	5
32	Friends encourage me to be physically active	□	□	□	□	□
33	Friends encourage me to eat healthy foods	□	□	□	□	□
34	Friends keep me company when I'm physically active	□	□	□	□	□
